# The Serum Protein Profile and Acute Phase Proteins in the Postoperative Period in Sheep after Induced Articular Cartilage Defect

**DOI:** 10.3390/ma12010142

**Published:** 2019-01-03

**Authors:** Csilla Tothova, Xenia Mihajlovicova, Jaroslav Novotny, Oskar Nagy, Maria Giretova, Lenka Kresakova, Marek Tomco, Zdenek Zert, Zuzana Vilhanova, Maros Varga, Lubomir Medvecky, Eva Petrovova

**Affiliations:** 1 Clinic of Ruminants, University of Veterinary Medicine and Pharmacy in Kosice, Komenskeho 73, 041 81 Kosice, Slovakia; Csilla.Tothova@uvlf.sk (C.T.); xenia.mihajlovicova@gmail.com (X.M.); Oskar.Nagy@uvlf.sk (O.N.); 2 Clinic of Swine, University of Veterinary Medicine and Pharmacy in Kosice, Komenskeho 73, 041 81 Kosice, Slovakia; Jaroslav.Novotny@uvlf.sk; 3 Institute of Materials Research SAS in Kosice, Watsonova 47, 040 01 Kosice, Slovakia; mgiretova@saske.sk (M.G.); lmedvecky@saske.sk (L.M.); 4 Institute of Anatomy, University of Veterinary Medicine and Pharmacy in Kosice, Komenskeho 73, 041 81 Kosice, Slovakia; lenka.kresakova@uvlf.sk (L.K.); mtomco.75@gmail.com (M.T.); 5 Clinic of Horses, University of Veterinary Medicine and Pharmacy in Kosice, Komenskeho 73, 041 81 Kosice, Slovakia; Zdenek.Zert@uvlf.sk (Z.Z.); z.vilhanova@gmail.com (Z.V.); 6 Sport-Arthro Centre, Privat Hospital Kosice-Saca, Lucna 57, 040 15 Kosice-Saca, Slovakia; maros.varga@nemocnicasaca.sk

**Keywords:** articular cartilage defect, bioplolymers, C-reactive protein, haptoglobin, in vivo testing, serum amyloid A, serum protein fractions, sheep

## Abstract

Although several new implants have been developed using animal studies for the treatment of osteochondral and cartilage defects, there is a lack of information on the possible metabolic and biochemical reactions of the body to the implantation of biomaterials and cartilage reconstruction. Therefore, this study was aimed at evaluating the serum protein pattern and the alterations in the concentrations of selected acute phase proteins in five clinically healthy female sheep before and after the reconstruction of experimentally induced articular cartilage defects using polyhydroxybutyrate/chitosan based biopolymer material. The concentrations of total serum proteins (TSP), protein fractions, and selected acute phase proteins—serum amyloid A (SAA), haptoglobin (Hp), and C-reactive protein (CRP)—were measured before and on days seven, 14, and 30 after the surgical intervention. The TSP concentrations showed no marked differences during the evaluated period. Albumin values decreased on day seven and day 14 after surgery. In the concentrations of α_1_-, α_2_-, β-, and γ_2_-globulins, a gradual significant increase was observed during the postoperative period (*p* < 0.05). The γ_1_-globulins decreased slightly seven days after surgery. The concentrations of SAA, Hp, and CRP increased significantly after the surgical intervention with a subsequent decrease on day 30. Presented results suggest marked alterations in the serum protein pattern after surgical intervention.

## 1. Introduction

The synthesis of serum proteins is strongly controlled to maintain their physiological balanced concentrations. Any pathological processes in the body may result in alterations in the serum protein concentrations [1]. In general, animals react to infection, inflammatory processes, trauma, or any disturbances in their homeostasis with a series of physiological, metabolic, and biochemical reactions known as the acute phase response [2]. The most important metabolic changes during the inflammatory responses include the highly increased or decreased production of some serum proteins, especially the acute phase proteins [3]. Due to their low physiological concentrations and high response in affected animals, they may serve as useful biomarkers for the evaluation of an animal’s health, clinical monitoring of different diseases, treatment responses, and prognostic purposes. In ruminants, serum amyloid A (SAA) and haptoglobin (Hp) are the diagnostically most important acute phase proteins [4]. C-reactive protein (CRP) has been described as a constitutive protein in these animal species, with only a minor increase during disease processes [5]. Nevertheless, the usefulness of CRP in the laboratory diagnosis of mastitis was evaluated by Schrodl et al. [6], and they found approximately 10-fold higher values in cows with mastitis compared with healthy ones.

In vivo animal studies are essential as a gap between in vitro experiments and human clinical studies for introducing biomaterials treatment into clinical orthopedic practice. Animal models are widely used in the research of innovative biomaterials for regeneration of articular cartilage defects. Mainly, large animal models with thicker articular cartilage permit the study of partial thickness and full thickness chondral repair, as well as osteochondral repair [7]. Anatomical location, size of the defect (critical or non-critical sized), as well as the mechanobiology, species, strain, age, and health conditions provide the highest scientifically relevant output related to the study aim, hypotheses, and direct translation to animal benefit [8]. The sheep is a commonly utilized animal model, as they are readily available, easy to handle, and are relatively inexpensive. Some unique features and a comparative description of the surgical anatomy and approaches to the stifle joint in sheep was published; it addressed the presence of the extensor digitorum longus muscle on the craniolateral aspect of the stifle joint, the absence of a cranial menisco-femoral ligament in the caudal joint space, and an attachment of the patellar tendon to the cranial pole of the patella when compared to man. Therefore, this joint may be considered by researchers who increasingly use sheep for studies on the replacement of cruciate ligaments, collateral ligaments and menisci, treatment of chondral and osteochondral defects, and osteoarthrosis [9]. The location of the cartilage defects in the ovine model has involved the medial femoral condyle and femoral trochlea as well, with a 7 mm reported critical size defect [10,11,12]. However, the selection of a suitable preclinical model for performance evaluation remains a challenge, as no gold standard exists to define the best animal model [8,13].

On the other side, there is a lack of information on the possible effects of cartilage reconstruction using biopolymers on the concentrations of biochemical indicators in animals. Alterations in the biomarker profile, including some serum proteins, may potentially indicate risk for the progression of the disease process and could be useful for early diagnosis of postoperative complications and infections in order to earlier detect uncontrolled inflammatory reactions and prevent prolonged convalescence [14]. Therefore, the aim of this study was to evaluate the alterations in the serum protein pattern and the concentrations of selected acute phase proteins in sheep after the reconstruction of experimentally induced articular cartilage defects using polyhydroxybutyrate/chitosan based biopolymer material, and to monitor their changes during the first 30 postoperative days.

## 2. Materials and Methods

### 2.1. Preparation and Characterization of Biopolymer Composite Implants

The polyhydroxybutyrate/chitosan blend (PHB/CHIT) was prepared according to Medvecky et al. [15]. Briefly, polyhydroxybutyrate (GoodFellow, dissolved in propylene carbonate) and chitosan (SigmaAldrich, Saint Louis, MO, USA, middle, dissolved in 1% acetic acid) were mutually mixed at a ratio equal to 1:1. The same volumes of differently concentrated biopolymer solutions were used for the precipitation. Mixing was carried out using a magnetic stirrer at 400 rpm. After 10 min of mixing, the acetone was slowly added to suspensions for the complete precipitation of biopolymers. Final blends were filtered, washed with distilled water, and molded into cylinder form (10 mm in diameter and 10 mm in height) and lyophilized (Ilshin) for 6 h. The microstructure of scaffolds was observed by scanning electron microscopy (FE SEM JEOL 7000, JEOL, Akishima, Tokyo). Implants were sterilized in an autoclave at 121 °C.

**Figure 1:**
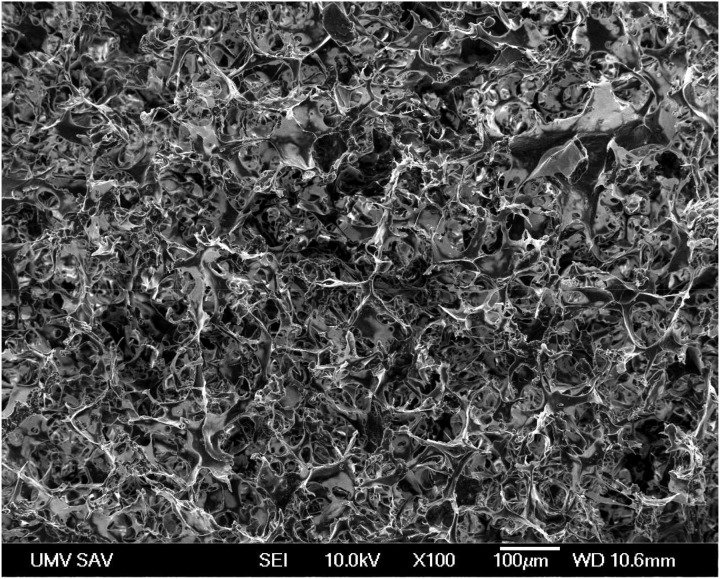
Scanning electron micrograph image shows the microstructure and presence of microporosity in the composite implant.

A macroporous microstructure of the spongy-like biopolymer composite implant with the high fractions of irregularly shaped macropores with sizes up to 100 μm and micropores (<20 µm) was observed in the chitosan/polyhydroxybutyrate blend (Figure 1). The biopolymers created fiber- and plate-like interconnected networks with open structures, which could be appropriate for both the migration of cells into the inner structure of the scaffold and the diffusion of body fluids (or metabolites) into or out of the implant.

### 2.2. Animals and Sample Collection

Approval of the experimental protocol was obtained from the State Veterinary and Food Administration of the Slovak Republic No. 3508/17-221. The study was carried out on five clinically healthy female sheep of the crossbreed of Merino and Valachian sheep (group E) from a farm PD Agro Michalovce (Michalovce, Slovakia) that was approved by the State Veterinary and Food Administration of the Slovak Republic. The animals were at the age of 1.5–2 years and in good nutritional condition with an average body weight of 50.7 ± 1.9 kg at arrival. They were housed on the Clinic of Ruminants of the University of Veterinary Medicine and Pharmacy in Kosice, Slovak Republic, in free-stalls with free access to water, hay, and concentrates during the time under study.

The animals were included into the study 30 days before the scheduled day of surgical intervention, allowing acclimatization to the changed environment. Before the inclusion into the study, the animals underwent standard preoperative clinical examinations. Clinical examinations included the assessment of the overall health status of the animals (food intake, behavior), inspection and recording of body temperature, respiratory and pulse rates, and a detailed evaluation of the organ systems [16]. After surgical intervention, the health status of the animals was evaluated daily until the end of the study, and was oriented to the observation of general health state after the surgical procedure, as well as local signs of inflammation in the surgical wound (heat, swelling, pain, discharge).

Blood samples for the determination of the concentrations of total serum proteins, selected acute phase proteins, and separation of serum protein fractions were obtained before surgery and on days 7, 14, and 30 after surgical intervention. To evaluate the changes in the concentrations of acute phase proteins in animals with induced articular cartilage defects, but without implantation of biopolymer, five clinically healthy sheep of the same age and breed were included into the study as a control group (group C). Blood samples were collected from the jugular vein into serum gel separator tubes without additives and anticoagulants (Meus, Piove di Sacco, Italy). Serum was separated after letting blood samples coagulate at room temperature and was then centrifuged at 3000× *g* for 30 min. The harvested serum was dispensed into plastic tubes and stored at −20 °C until it was analyzed.

### 2.3. Surgical Procedure

**Figure 2:**
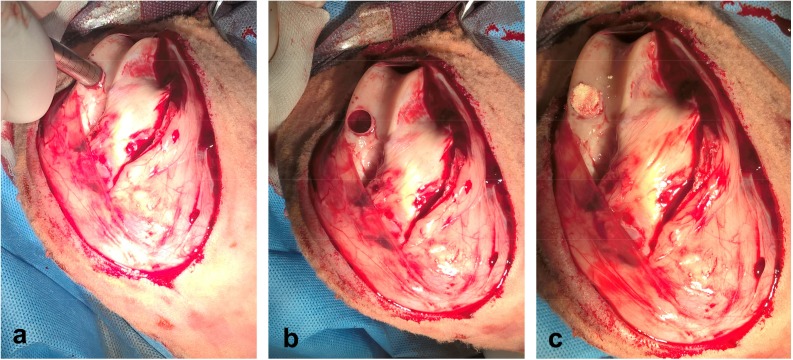
Surgical procedure: (**a**) inducing of articular cartilage defect with OATS equipment (Osteochondral autograft transfer system, Arthrex, Naples, FL, USA); (**b**) preparing articular cartilage defect in femoral trochlea before implantation; (**c**) inserting of scaffold into prepared articular cartilage defect.

**Figure 3:**
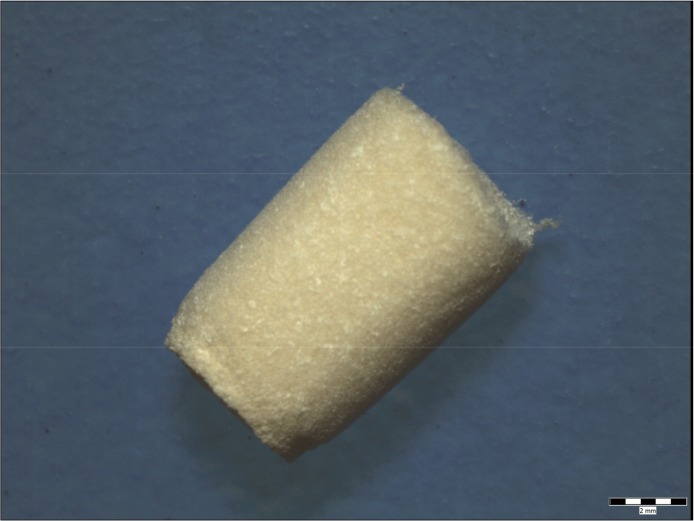
Macrostructure of scaffold before implantation. Scale bar: 2 mm.

Food and water in the used animals were withheld for 12 h before the surgery. Anesthesia consisted of a mixture of buthorphanol (0.1 mg/kg, Butomidor 10 mg/mL, Richter Pharma, Wels, Austria), and medetomidin 0.02 mg/kg (Cepetor 1 mg/mL, CP-Pharma Handelsgesellschaft, GmbH, Burgdorf, Germany) administered intramuscularly, and ketamin 8 mg/kg (Ketamidor 100 mg/mL, Richter Pharma, Wels, Austria) administered intravenously. After anesthesia, a defect in the articular cartilage of the left stifle joint was induced. An incision was made from the left lateral side, from the medial patellar ligament distal to the tibial tuberosity. The stifle joint was visualized above the medial femoral condyle load. The subcutaneous tissue and superficial fascia were incised. After flexion and partial luxation of the stifle joint using the Osteochondral autograft transfer system (Arthrex, Naples, FL, USA), a defect was made on the articular cartilage on the exact place in the distal epiphysis of the femur (*trochlea femoris sinister* [17]) at a diameter of 10 mm and a depth of 10 mm (Figure 2). The site of the created defect was then filled with a biopolymer implant (Figure 3), which was in vitro tested for cytotoxicity [18]. The same procedure was used to create a defect in the animals from the control group, but it was not filled with biopolymer material. All sheep received postoperative systemic broad spectrum antibiotic oxytetracyclinum dihydricum 20 mg/kg (Alamycin LA a.u.v., Norbrook, Newry, UK, once every second day) and non-steroidal anti-inflammatory drug flunixin meglumine 2.2 mg/kg (Flunixin a.u.v., Norbrook, Newry, UK, once a day), administered intramuscularly for 7 days.

### 2.4. Laboratory Analyses

The total serum protein (TP, g/L) concentrations were assessed according to the Biuret method on an automated biochemical analyzer Alizé (Lisabio, Poully en Auxois, France) using commercial diagnostic kits (TP 245, Randox, Crumlin, UK). The serum protein fractions were separated by zone electrophoresis on an agarose gel using an automated electrophoresis system Hydrasys (Sebia Corporate, Lisses, Evry Cedex, France) with commercial diagnostic kits Hydragel 7 Proteine (PN 4100, Sebia Corporate, Lisses, Evry Cedex, France) according to the procedure described by the manufacturer. The densitometry scanning system Epson Perfection V700 (Epson America Inc., Long Beach, CA, USA) was used to scan the electrophoretic gels based on the method of light transmission and conversion into an optical density curve. The gel images were visualized using the computer software Phoresis version 5.50 (Sebia Corporate, Lisses, Evry Cedex, France). The following protein fractions were identified: albumin, α_1_- and α_2_-globulins, β-globulins, and γ_1_- and γ_2_-globulins. Each protein fraction was expressed as relative concentrations (%) according to the obtained optical density. Consequently, their absolute concentrations (g/L) were quantified from the total serum protein concentrations. The ratios of albumin to globulins (A/G) were calculated as well.

The serum concentrations of selected inflammatory markers—serum amyloid A (SAA; mg/mL), Hp (mg/mL), and CRP (μg/mL)—were measured to evaluate the postoperative inflammatory state. SAA was analyzed by sandwich enzyme linked immunosorbent assay (ELISA) using commercial multispecies kits (TP-802, Tridelta Developmet, Kildare, Ireland). Sheep CRP was measured by solid-phase ELISA assay using commercially available tests (CRP-12, Life Diagnostics, Inc., West Chester, PA, USA). Haptoglobin was assessed using commercial colorimetric kits (TP-801, Tridelta Development, Kildare, Ireland) in microplates based on Hp-haemoglobin binding and the preservation of the peroxidase activity of the bound haemoglobin at a low pH. The absorbances were read on the automatic microplate reader Opsys MR (The Dynex Technologies, Chantilly, VA, USA). The results were calculated using the computer software Revelation QuickLink version 4.25 (Dynex Technologies, Chantilly, VA, USA).

### 2.5. Statistical Analyses

Descriptive statistical procedures were used to calculate arithmetic means (x) and standard deviations (SD) for each evaluated variable and sample collection time. The distribution of data was evaluated by the Kolmogorov-Smirnov test for normality. Not all of the obtained data passed the normality test. Therefore, repeated-measures one-way ANOVA was used to examine the changes during the perioperative period for normally distributed data with equal variance, and the Friedman test was used for non-normally distributed data. The significance of differences in values between the sample collections was evaluated by Tukey-Kramer and Dunn`s Multiple Comparisons tests. For the analysis of the acute phase protein concentrations, two-way repeated measures ANOVA was used. All statistical analyses were carried out using the program GraphPad Prism V5.02 (GraphPad Software Inc., San Diego, CA, USA).

## 3. Results

Various grades of lameness were observed in all sheep for 90 days after the surgical intervention. The surgical wound developed no signs of inflammation and was without discharge in all the evaluated sheep. The animals showed improvement with no serious complications and inflammatory processes in other organ systems.

**Figure 4:**
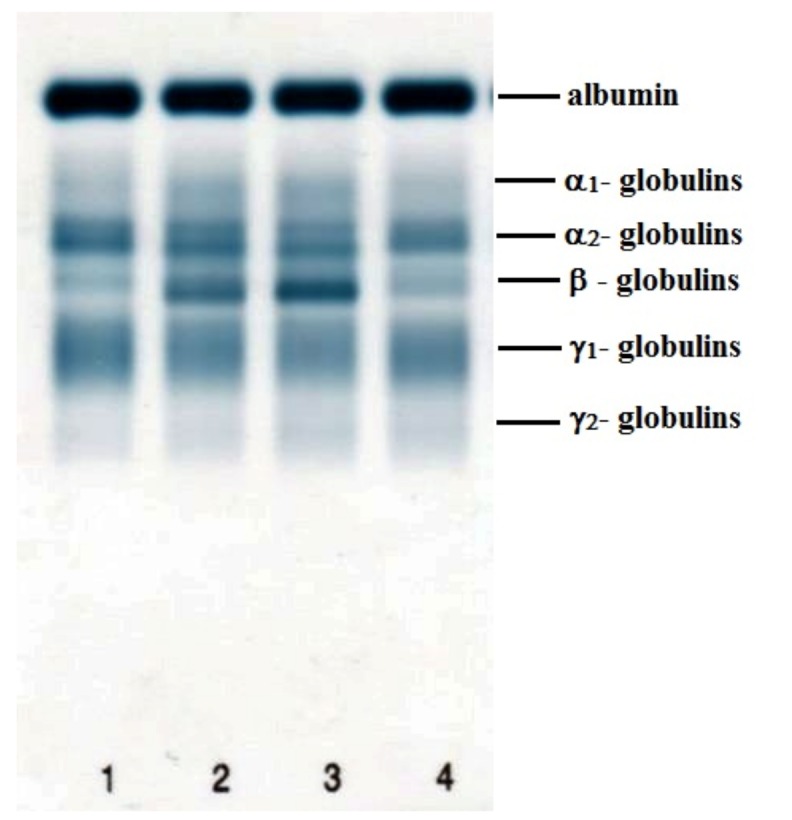
Example of the electrophoretic separation of serum proteins using agarose gel electrophoresis in a sheep during the perioperative period: (**1**) before surgical intervention; (**2**) day 7; (**3**) day 14; (**4**) day 30 after surgery.

**Figure 5:**
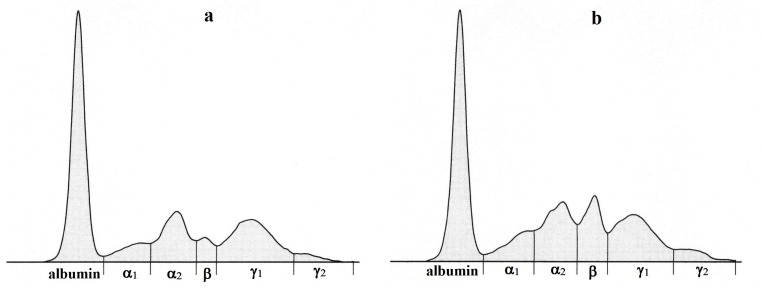

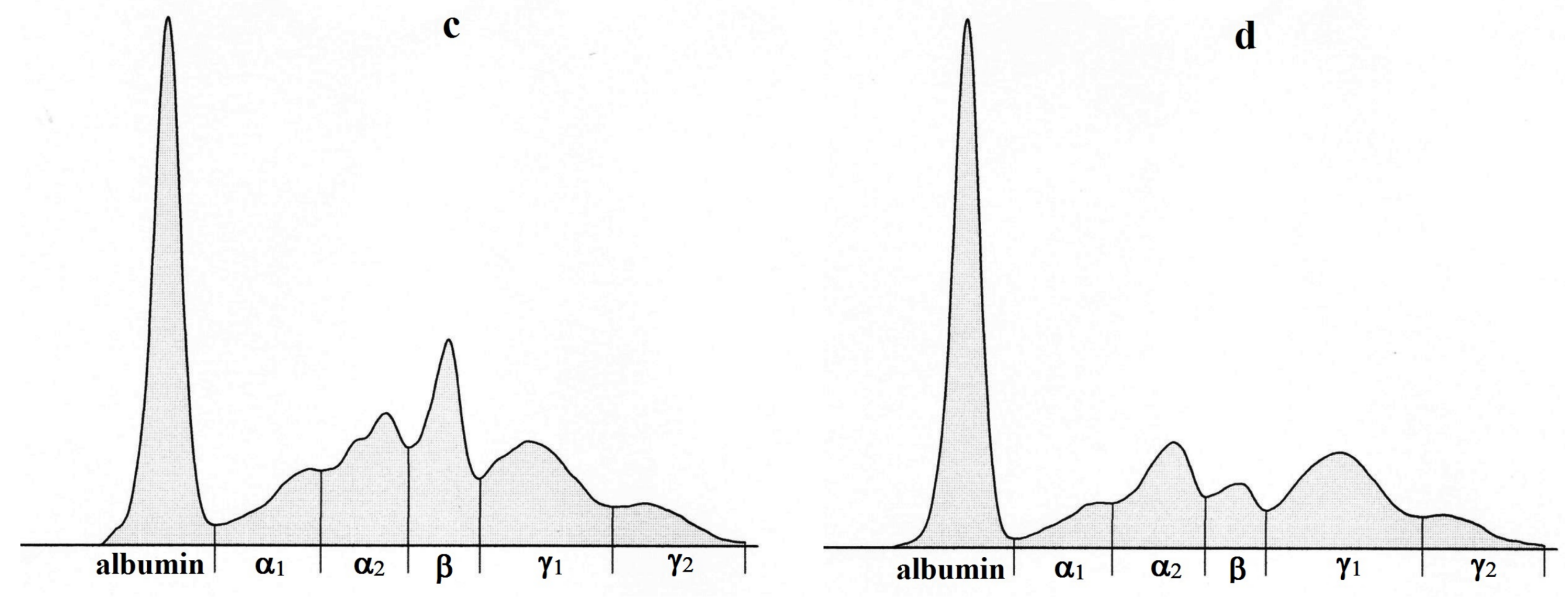
Representative electrophoretograms in a sheep showing the protein fractionation into six fractions: albumin, α_1_-, α_2_-, β-, γ_1_-, and γ_2_-globulins before surgical intervention (**a**) and in the postoperative period-7 (**b**), 14 (**c**) and 30 days (**d**) after surgery.

The data obtained during the perioperative period are presented in Table 1, Table 2 and Table 3. The electrophoretic separation of serum proteins using agarose gel is presented in Figure 4. Figure 5 shows representative examples of electrophoretograms before the surgical intervention and in the postoperative period.

The relative concentrations of albumin (Table 1) showed in sheep a marked decrease seven days after the surgical intervention with a further slight decrease on day 14 and a subsequent increase on day 30 after surgery. However, the changes of albumin values during the evaluated period were not significant. Significant alterations during the perioperative period were observed in the relative concentrations of α_1_-globulins (*p* < 0.05). Their values increased seven days after surgery, stayed relatively stable, and then slightly decreased on day 30. In the relative concentrations of α_2_-globulins, a significantly higher mean value was found seven days after surgery (*p* < 0.05) with a tendency to further decrease to values comparable with those obtained prior to surgery. The relative concentrations of β-globulins increased gradually and significantly until day 14 after surgery (*p* < 0.01) with a subsequent slight decrease on day 30 of the postoperative period. The relative concentrations of γ_1_-globulins were relatively stable during the evaluated period. On the other hand, the relative values of γ_2_-globulins increased significantly until day 30 after surgery (*p* < 0.05). An opposite trend was observed in the A/G ratios, which were the highest prior to surgery. The values obtained after the surgical procedure were markedly lower.

No marked differences were found in the concentrations of total serum proteins (Table 2) before surgery and in the early postoperative period. A slight non-significant increase of values was recorded on day 14 after surgery with a subsequent decrease on the end of the evaluated period. The highest mean absolute concentration of albumin was obtained prior to surgery. The values obtained in the postoperative period were non-significantly lower. Significant alterations during the evaluated period were found in the absolute concentrations of α_1_-globulins (*p* < 0.05), showing a gradual significant increase until day 14 after surgery (*p* < 0.05) and a subsequent decrease on day 30 of the postoperative period. Similar trends of gradually increasing values until day 14 after the surgical intervention were observed in the absolute concentrations of α_2_- and β-globulins (*p* < 0.05). On the other hand, the absolute concentrations of γ_1_-globulins decreased slightly seven days after surgery with a subsequent gradual increase to preoperative values. In the absolute concentrations of γ_2_-globulins, a gradual significant increase of mean values was found until the end of the evaluated period (*p* < 0.05).

The evaluation of the concentrations of SAA in the period before and after surgery showed significant changes in both the experimental and the control group of sheep (*p* < 0.01, *p* < 0.05, respectively, Table 3). In the experimental group, the values obtained before surgery were low and increased significantly in response to the surgical procedure, being the highest on day seven after surgery (*p* < 0.01). From day 14 after surgery, a gradual decrease of values was found up to day 30 of the postoperative period. In the control group of sheep, only a slight increase of values was observed seven days after surgical intervention with a repeated gradual decrease until the end of the evaluated period. In the concentrations of Hp in the experimental group of sheep, a more gradual non-significant increase was found until day 14 after surgery with a subsequent decrease on day 30 after the surgical procedure. The mean value obtained on day 30 after surgery was more than 20-fold higher than the preoperative concentrations. In the control group of sheep, the values increased less markedly and non-significantly up to day 14 after the surgical intervention and then decreased. Regarding the concentrations of CRP in the experimental group, a slight gradual significant increase of values was observed, these values being the highest on day 14 after surgery (*p* < 0.05) and then starting to decrease. These changes during the evaluated period were significant (*p* < 0.05). The concentrations of CRP in the control group showed a slight non-significant increase seven days after surgery with a subsequent gradual decrease until the end of the evaluated period.

## 4. Discussion

Animal studies are fundamental to showing the efficacy and safety of new cartilage defects repair before its clinical use in humans. Not only small experimental animals, but also large animal models (goats, sheep, pigs) have been successfully used to demonstrate new implants for the treatment of osteochondral and cartilage defects [11,19,20]. However, these studies were predominantly oriented to the histological evaluation of the cartilage repair and maturation of the newly-formed cartilage. The metabolic and biochemical reactions of the body to the implantation of biomaterials and cartilage reconstruction have not been studied in depth. The results of the presented study showed in sheep some marked biochemical responses to the articular cartilage defect repair characterized by alterations in the serum protein electrophoretic pattern and in the production of the evaluated acute phase proteins.

The analyses of the total serum protein concentrations showed in sheep no marked changes after the induction and reconstruction of the cartilage defect when compared with the values obtained before the intervention. A slight increase of total protein concentrations was recorded on day 14 after the surgical intervention, probably related to the response of the organism to the damage caused in the articular cartilage. The patterns of proteins in the serum or synovial fluid, their fractionation, and the identification of several proteins have been studied in some cartilage lesions, joint disorders, and destruction in both humans and animals [21,22,23]. However, the profile of serum proteins after cartilage reconstruction and implantation of biomaterials was previously not described. In the presented study, major alterations were observed in the electrophoretic pattern of serum proteins and the distribution of most of the serum protein fractions. In the serum albumin concentrations, a trend of lower values was observed after the creation of cartilage defect and implantation of biopolymer. Its values started to increase on day 30 of the postoperative period. Albumin is a major negative acute phase protein. Therefore, its decreased concentrations after the surgical intervention might be attributed to this function of albumin [24]. Whitaker et al. [25] stated that albumin is one of metabolic parameters that may be used to monitor inflammatory diseases. According to Hübner et al. [26], the decrease in the concentrations of albumin in early post operation may reflect the magnitude of surgical trauma and predict adverse clinical outcomes.

An opposite trend was observed in the concentrations of α-globulins (α_1_-, as well as α_2_-globulins). Their values increased until day 14 after surgical intervention, then started to decrease, and finally returned to preoperative concentrations 30 days after surgery. The alpha-globulins constitute a large fraction on the electrophoretogram, which is composed of many diagnostically important proteins. Furthermore, some of them act as acute phase proteins that play different roles in the host defense responses, regulation of inflammatory processes, and restoration of homeostasis [27]. While alpha_1_-antitrypsin, α_1_-acid glycoprotein, α_1_-antichymotrypsin, α_1_-fetoprotein, serum amyloid A, and α_1_-lipoprotein belong to the α_1_-globulin fraction, haptoglobin, α_2_-microglobulin, α_2_-macroglobulin, ceruloplasmin, α_2_-antiplasmin, and α_2_-lipoprotein migrate in the α_2_-globulin fraction [28]. Thus, the increases of the alpha fractions after surgery may reflect the increases in the concentrations of some acute phase proteins resulting from the activation of the host inflammatory responses due to the damage caused in the articular cartilage and its reconstruction. The concentrations of SAA and Hp in the evaluated sheep were low before surgery and increased after the induction of cartilage damage and implantation of polyhydroxybutyrate/chitosan based biopolymer. On day 30 after implantation, the values showed a tendency to decrease to those obtained prior to surgery, suggesting an uncomplicated postoperative period and the absence of inflammatory reactions. Differences were obtained in the rate of increase and subsequent decrease during the postoperative period among the measured inflammatory markers as well as between the experimental and control group. While the concentrations of SAA in the experimental sheep increased more than 200-fold already seven days after the surgical intervention, the values of Hp showed a more gradual increase until day 14 after surgery (approximately a 50-fold increase). On the other hand, the concentrations of SAA in the control group increased approximately 1.5-fold seven days after surgery, and the values of Hp increased about 13-fold up to day 14 after the surgery. SAA is a sensitive biomarker characterized by remarkable increase and rapid decrease once the inflammatory stimuli are eliminated. On the other hand, Hp is characterized by a more prolonged response and is thus preferable in the field to evaluate disease processes [27]. Aulin et al. [29] found in rabbits a pronounced increase of SAA concentrations after the surgical induction of full thickness osteochondral defects in the femorotibial joints. Within one-month postoperatively, the SAA values returned to physiological concentrations, supporting the absence of inflammation. In contrast, in cases with ongoing infection, persistently high or even increased SAA values can be observed, suggesting complications after surgery or indicating a lack of treatment response [30]. Thus, the trend of decreasing SAA and Hp values observed in our study may indicate an uncomplicated postoperative period. However, seeing that there are scarce literature data about the usefulness of these biomarkers in the evaluation of cartilage regeneration, further studies would be helpful.

A similar increasing trend was found in the concentrations of β-globulins until day 14 after surgery, showing a markedly higher and narrow peak on the electrophoretogram. After this period, their values started to decline. The C-reactive protein was identified in the β-globulin fraction. Its concentrations in sheep were relatively high prior to surgery and increased only slightly after the cartilage damage. Higher CRP values were found in patients with synovial inflammation and cartilage damage due to femoroacetabular impingement [31,32]. However, little is known about the behavior of CRP after cartilage reconstruction using biomaterial implants and during the postoperative period. On the other hand, some other proteins may be identified in the β-globulin fraction, including complement, transferrin, ferritin, as well as β_2_-microglobulin or β-lipoproteins. These proteins are involved in the regulation of inflammatory processes and stress responses and thus may be attributed to the marked elevation of the β-globulin fraction after the induction of cartilage damage [33]. Thirty days after the surgical intervention, their concentrations started to decrease, reflecting no serious complications during the treatment.

The γ-globulin fraction is predominantly composed of immunoglobulins (Ig) of various classes (IgG, IgA, IgM, IgD, and IgE). Sheep are a typical example of an animal species in which γ-globulins may be separated into two subfractions (γ_1_ and γ_2_) [34]. In the presented study, the concentrations of γ_1_-globulins decreased slightly seven days after surgery and subsequently started to increase to preoperative values. On the other hand, the values of γ_2_-globulins showed a gradual increase until the end of the evaluated period. Most of the immunoglobulin classes migrate in the γ_1_-globulin fraction, but some IgG subclasses (the so called slow immunoglobulins) may be detected in the γ_2_-globulin fraction and generally have antibody activity [35]. Thus, the increase of γ_2_-globulin concentrations in sheep after surgery might reflect the response of the organism to the damage caused by surgical intervention and the implantation of biomaterial. Further studies would be helpful in yielding satisfactory results. The changes observed in the concentrations of albumin and globulin fractions also resulted in alterations in the A/G ratio. The highest A/G ratio was observed in sheep before surgery, and the values obtained after the intervention were lower due to the overproduction of globulins caused by the cartilage damage and its repair.

The dynamics of changes in the concentrations of the evaluated biomarkers might be influenced by the degree of cartilage damage, as well as surgical stress, general anesthesia, the course of regeneration, and antimicrobial and anti-inflammatory treatments during the postoperative period [36,37]. Stowasser-Raschbauer et al. [38] concluded that the rise of the concentrations of SAA in horses after surgical procedures under general anesthesia is partly due to the anesthetic procedure. In horses, an increase in SAA concentrations was found after general anesthesia but without surgery, while the increase was higher when surgery was performed. On the other hand, Pepys et al. [39] reported in horses no increase in the concentrations of SAA after general anesthesia. However, there are no published reports about the effect of general anesthesia on the concentrations of acute phase protein in farm animals. Similar contradictory data were published regarding the effect of non-steroidal anti-inflammatory drugs on the acute phase response during the postoperative period and recovery. Ting et al. [40] concluded that repeated administration of ketoprofen in beef cattle after surgery did not have influence on the changes in acute phase protein concentrations when compared with non-treated animals. On the other hand, Plessers et al. [41] suggested that non-steroidal anti-inflammatory drug (NSAID) ketoprofen may attenuate the acute phase response in calves, as they found lower concentrations of inflammatory markers, including SAA, after the administration of ketoprofen in calves challenged by lipopolysaccharide. Similar to studies in humans, differences in the reactivity and variability of individual animals may account for differences in the responses to the induction of cartilage defects and their reconstruction, consequently resulting in a wider range of not uniform and inconsistent data [7]. Therefore, larger experimental animal groups are needed to obtain further results in cartilage repair studies.

## 5. Conclusions

In conclusion, the presented results suggest marked alterations in the serum protein pattern in sheep with surgically induced articular cartilage defects, which are characterized by changes in most of the serum proteins fractions and acute phase protein concentrations. After cartilage repair and approximately four weeks after the implantation, the concentrations decreased to preoperative values, suggesting the absence of further inflammatory reactions during the postoperative period. These results indicate that some biomarkers from the serum protein profile may be used for the evaluation of the postoperative period, the progression of the disease process, and the uncontrolled postoperative inflammatory responses. However, further comprehensive studies completed with the examination of the postoperative period by further blood parameters and made with more dense animal groups are needed to yield satisfactory results.

## Figures and Tables

**Table 1:** Changes in the relative concentrations of serum protein fractions (%) and albumin/globulin ratio (A/G) in sheep before surgical intervention and in the postoperative period (mean ± SD).

Variables	Sample Collection				*p* Value
	Before Surgical Intervention	7 Days After Surgical Intervention	14 Days After Surgical Intervention	30 Days After Surgical Intervention	
Albumin	55.2 ± 8.9	45.3 ± 8.1	41.0 ± 8.9	45.8 ± 4.9	n.s.
α_1_-globulins	6.0 ± 0.7 ^a^	8.3 ± 2.2	8.3 ± 1.3 ^a^	7.0 ± 0.8	<0.05
α_2_-globulins	13.5 ± 1.2 ^a^	16.2 ± 1.2 ^a^	15.6 ± 1.5	13.5 ± 1.3	<0.001
β-globulins	4.2 ± 1.1 ^A^	9.7 ± 4.9	13.4 ± 7.0 ^A^	8.8 ± 2.6	<0.01
γ_1_-globulins	18.1 ± 6.6	16.7 ± 5.5	17.0 ± 4.1	19.0 ± 3.1	n.s.
γ_2_-globulins	2.9 ± 0.7 ^a^	3.8 ± 1.4	4.7 ± 0.8	5.8 ± 1.6 ^a^	<0.05
A/G	1.30 ± 0.45	0.86 ± 0.31	0.73 ± 0.30	0.86 ± 0.16	n.s.

The superscripts in the same rows mean statistically significant differences between the sample collections (a—*p* < 0.05, A—*p* < 0.01); *p* value—significance of the statistical tests; n.s.—not significant.

**Table 2:** Changes in the absolute concentrations of total serum protein (TP), and protein fractions (g/L) in sheep before surgical intervention and in the postoperative period (mean ± SD).

Variables	Sample Collection				*p*-Value
	Before Surgical Intervention	7 Days After Surgical Intervention	14 Days After Surgical Intervention	30 Days After Surgical Intervention	
TP	65.7 ± 7.1	64.4 ± 4.2	68.2 ± 1.6	64.7 ± 2.1	n.s.
Albumin	36.0 ± 4.7	31.0 ± 4.5	27.9 ± 6.0	29.6 ± 3.0	n.s.
α_1_-globulins	4.0 ± 0.8 ^a^	5.4 ± 1.5	5.7 ± 0.9 ^a^	4.5 ± 0.5	<0.05
α_2_-globulins	8.9 ± 1.4	10.4 ± 0.6	10.6 ± 0.9	8.7 ± 0.6	<0.05
β-globulins	2.8 ± 1.0 ^a^	6.3 ± 3.1	9.1 ± 4.8 ^a^	5.7 ± 1.8	<0.05
γ_1_-globulins	12.2 ± 5.5	10.8 ± 4.2	11.6 ± 3.0	12.3 ± 2.2	n.s.
γ_2_-globulins	1.9 ± 0.7^a^	2.5 ± 1.1	3.2 ± 0.5	3.7 ± 1.1 ^a^	< 0.05

The superscripts in the same rows mean statistically significant differences between the sample collections (a—*p* < 0.05); *p* value—significance of the statistical tests; n.s.—not significant.

**Table 3:** Changes in the concentrations of SAA, Hp and CRP in the experimental (E) and control (C) group of sheep before surgical intervention and in the postoperative period (mean ± SD).

Variables		Sample Collection				*p* Value
		Before Surgical Intervention	7 Days After Surgical Intervention	14 Days After Surgical Intervention	30 Days After Surgical Intervention	
SAA (μg/mL)	E	0.73 ± 1.14 ^A^	154.40 ± 83.67 ^A‡^	47.27 ± 55.21	8.84 ± 7.49 ^†^	<0.01
	C	3.99 ± 6.28	5.62 ± 6.67 ^a‡^	1.27 ± 1.18	0.28 ± 0.31 ^a†^	<0.05
Hp (mg/mL)	E	0.188 ± 0.019 ^‡^	6.306 ± 7.417	10.330 ± 9.413 ^†^	4.250 ± 5.295	n.s.
	C	0.061 ± 0.071 ^‡^	0.190 ± 0.284	0.831 ± 1.140 ^†^	0.112 ± 0.094	n.s.
CRP (μg/mL)	E	147.8 ± 43.52 ^a^	189.0 ± 49.51	259.6 ± 75.71 ^a†^	187.2 ± 51.12	<0.05
	C	202.8 ± 128.3	222.8 ± 127.3	172.5 ± 60.1 ^†^	145.4 ± 44.7	n.s.

The superscripts in the same rows mean statistically significant differences between the sample collections (a—*p* < 0.05, A—*p* < 0.01); the superscripts in the same columns of each variable mean statistically significant differences between the experimental and control group (†—*p* < 0.05, ‡—*p* < 0.01); *p* value—significance of the statistical tests; n.s.—not significant.

## References

[1] Pieper R., Gatlin C.L., Makusky A.J., Russo P.S., Schatz C.R., Miller S.S., Su Q., McGrath A.M., Estock M.A., Parmar P.P. (2003). The human serum proteome: Display of nearly 3700 chromatographically separated protein spots on two-dimensional electrophoresis gels and identification of 325 distinct proteins. Proteomics.

[2] Ceciliani F., Ceron J.J., Eckersall P.D., Sauerwein H. (2012). Acute phase proteins in ruminants. J. Proteom..

[3] Murata H., Shimada N., Yoshioka M. (2004). Current research on acute phase proteins in veterinary diagnosis: An overview. Vet. J..

[4] Eckersall P.D., Bell R. (2010). Acute phase proteins: Biomarkers of infection and inflammation in veterinary medicine. Vet. J..

[5] Eckersall P.D., Kaneko J.J., Harvey J.W., Bruss M.L. (2008). Proteins, proteomics, and the dysproteinemias. Clinical Biochemistry of Domestic Animals.

[6] Schrodl W., Kruger M., Hien T.T., Fuldner M., Kunze R. (1995). C-reactive protein as a new parameter of mastitis. Tierarztl. Prax..

[7] Chu C.R., Szczodry M., Bruno S. (2010). Animal models for cartilage regeneration and repair. Tissue Eng..

[8] Bongio M., van den Beucken J.J.J.P., Leeuwenburgh S.C.G., Jansen J.A. (2015). Preclinical evaluation of injectable bone substitute materials. J. Tissue Eng. Regen. Med..

[9] Martini L., Fini M., Giavaresi G., Giardino R. (2001). Sheep Model in Orthopedic Research: A Literature Review. Comp. Med..

[10] Ahern B.J., Parvizi J., Boston R., Schaer T.P. (2009). Preclinical animal models in single site cartilage defect testing: A systematic review. Osteoarthr. Cartil..

[11] Hao T., Wen N., Cao J.-K., Wang H.-B., Lü S.-H., Liu T., Lin Q.-X., Duan C.-M., Wang C.-Y. (2010). The support of matrix accumulation and the promotion of sheep articular cartilage defects repair in vivo by chitosan hydrogels. Osteoarthr. Cartil..

[12] Sidler M., Fouché N., Meth I., von Hahn F., von Rechenberg B., Kronen P.W. (2013). Transcutaneous Treatment with Vetdrop^®^ Sustains the Adjacent Cartilage in a Microfracturing Joint Defect Model in Sheep. Open Orthop. J..

[13] Li Y., Chen S.-K., Li L., Qin L., Wang X.-L., Lai Y.-X. (2015). Bone defect animal models for testing efficacy of bone substitute biomaterials. J. Orthop. Transl..

[14] Demura S., Takahashi K., Kawahara N., Watanabe Y., Tomita K. (2006). Serum interleukin-6 response after spinal surgery: Estimation of surgical magnitude. J. Orthop. Sci..

[15] Medvecky L., Giretova M., Stulajterova R. (2014). Properties and in vitro characterization of polyhydroxybutyrate–chitosan scaffolds prepared by modified precipitation method. J. Mater. Sci. Mater. Med..

[16] Jackson P.G.G., Cockcroft P.D. (2002). Clinical Examination of Farm Animals.

[17] Danko J., Simon F., Artimova J. (2011). Nomina Anatomica Veterinaria.

[18] Giretova M., Medvecky L., Stulajterova R., Sopcak T., Briancin J., Tatarkova M. (2016). Effect of enzymatic degradation of chitosan in polyhydroxybutyrate/chitosan/calcium phosphate composites on in vitro osteoblast response. J. Mater. Sci. Mater. Med..

[19] Hembry R.M., Dyce J., Driesang I., Hunziker E.B., Fosang A.J., Tyler J.A., Murphy G. (2000). Immunolocalization of matrix metalloproteinases in partial-thickness defects in pig articular cartilage. J. Bone Jt. Surg. Am..

[20] Niederauer G.G., Slivka M.A., Leatherbury N.C., Korvick D.L., Harroff H.H., Ehler W.C., Dunn C.J., Kieswetter K. (2000). Evaluation of multiphase implants for repair of focal osteochondral defects in goats. Biomaterials.

[21] Fujimura K., Segami N., Yoshitake Y., Tsuruoka N., Kaneyama K., Sato J., Kobayashi S. (2006). Electrophoretic separation of the synovial fluid proteins in patients with temporomandibular joint disorders. Oral Surg. Oral Med. Oral Pathol. Oral Radiol. Endodontol..

[22] Basile R.C., Ferraz G.C., Carvalho M.P., Albernaz R.M., Araújo R.A., Fagliari J.J., Queiroz-Neto A. (2013). Physiological concentrations of acute-phase proteins and immunoglobulins in equine synovial fluid. J. Equine Vet. Sci..

[23] Barrachina L., Remacha A.R., Soler L., Garcia N., Romero A., Vázquez F.J., Vitoria A., Álava M.Á., Lamprave F., Rodellar C. (2016). Acute phase protein haptoglobin as inflammatory marker in serum and synovial fluid in an equine model of arthritis. Vet. Immunol. Immunopathol..

[24] Gruys E., Obwolo M.J., Toussaint M.J.M. (1994). Diagnostic significance of the major acute phase proteins in veterinary clinical chemistry: A review. Vet. Bull..

[25] Whitaker D.A., Goodger W.J., Garcia M., Perera B.M.A.O., Wittwer F. (1999). Use of metabolic profiles in dairy cattle in tropical and subtropical countries on smallholder dairy farms. Prev. Vet. Med..

[26] Hübner M., Mantziari S., Demartines N., Pralong F., Coti-Bertrend P., Schäfer M. (2016). Postoperative albumin drop is a marker for surgical stress and a predictor for clinical outcome: A pilot study. Gastroenterol. Res. Pract..

[27] Petersen H.H., Nielsen J.P., Heegaard P.M.H. (2004). Application of acute phase protein measurements in veterinary clinical chemistry. Vet. Res..

[28] Bossuyt X. (2006). Advances in serum protein electrophoresis. Adv. Clin. Chem..

[29] Aulin C., Jensen-Waern M., Ekman S., Hägglund M., Engstrand T., Hilborn J., Hedenqvist P. (2013). Cartilage repair of experimentally 11 induced osteochondral defects in New Zealand White rabbits. Lab. Anim..

[30] Pollock P.J., Prendergast M., Schumacher J., Bellenger C.R. (2005). Effects of surgery on the acute phase response in clinically normal and diseased horses. Vet. Rec..

[31] Pearle A.D., Scanzello C.R., George S., Mandl L.A., DiCarlo E.F., Peterson M., Sculco T.P., Crow M.K. (2007). Elevated high-sensitivity C-reactive protein levels are associated with local inflammatory findings in patients with osteoarthritis 1. Osteoarthr. Cartil..

[32] Bedi A., Lynch E.B., Sibilsky Enselman E.R., Davis M.E., DeWolf P.D., Makki T.A., Kelly B.T., Larson C.M., Henning P.T., Mendias C.L. (2013). Elevation in circulating biomarkers of cartilage damage and inflammation in athletes with femoroacetabular impingement. Am. J. Sports Med..

[33] Bernabucci U., Lacetera N., Danieli P.P., Bani P. (2009). Influence of different periods of exposure to hot environment on rumen function and diet digestibility in sheep. Int. J. Biometeorol..

[34] Nagy O., Tóthová C., Nagyová V., Kováč G. (2015). Comparison of serum protein electrophoretic pattern in cows and small ruminants. Acta Vet. Brno.

[35] Kaneko J.J. (1997). Serum proteins and the dysproteinemias. Clinical Biochemistry of Domestic Animals.

[36] Jacobsen S., Nielsen J.V., Kjelgaard-Hansen M., Toelboell T., Fjeldborg J., Halling-Thomsen M., Martinussen T., Thoefner M.B. (2009). Acute phase response to surgery of varying intensity in horses: A preliminary study. Vet. Surg..

[37] Busk P., Jacobsen S., Martinussen T. (2010). Administration of perioperative penicillin reduces postoperative serum amyloid A response in horses being castrated standing. Vet. Surg..

[38] Stowasser-Raschbauer B., Kabeš R., Moens Y. (2013). Serum amyloid A concentrations in horses following anesthesis with and without surgery. Tierärztl. Monat. Vet. Med. Austria.

[39] Pepys M.B., Baltz M.L., Tennent G.A., Kent J., Ousey J., Rossdale P.D. (1989). Serum amyloid A protein (SAA) in horses: Objective measurement of the acute phase response. Equine Vet. J..

[40] Ting S.T.L., Earley B., Hughes J.M.L., Crowe M.A. (2003). Effect of ketoprofen, lidocaine local anesthesia, and combined xylazine and lidocaine caudal epidural anesthesia during castration of beef cattle on stress responses, imunity, growth, and behavior. J. Anim. Sci..

[41] Plessers E., Wyns H., Watteyn A., Pardon B., De Baere S., Sys S.U., De Backer P., Croubels S. (2016). Immunomodulatory properties of gamithromycin and ketoprofen in lipopolysaccharide-challenged calves with emphasis on the acute-phase response. Vet. Immunol. Immunopathol..

